# Polyamine Metabolism and Oxidative Protein Folding in the ER as ROS-Producing Systems Neglected in Virology

**DOI:** 10.3390/ijms19041219

**Published:** 2018-04-17

**Authors:** Olga A. Smirnova, Birke Bartosch, Natalia F. Zakirova, Sergey N. Kochetkov, Alexander V. Ivanov

**Affiliations:** 1 Engelhardt Institute of Molecular Biology, Russian Academy of Sciences, Vavilov str. 32, Moscow 119991, Russia; o.smirnova.imb@gmail.com (O.A.S.); nat_zakirova@mail.ru (N.F.Z.); kochet@eimb.ru (S.N.K.); 2 Cancer Research Center Lyon, INSERM U1052 and CNRS 5286, Lyon University, 69003 Lyon, France; birke.bartosch@inserm.fr; 3 DevWeCan Laboratories of Excellence Network (Labex), Lyon 69003, France

**Keywords:** reactive oxygen species, peroxide, polyamines, spermine, spermidine, spermine oxidase, oxidoreductin, oxidative protein folding, calcium

## Abstract

Reactive oxygen species (ROS) are produced in various cell compartments by an array of enzymes and processes. An excess of ROS production can be hazardous for normal cell functioning, whereas at normal levels, ROS act as vital regulators of many signal transduction pathways and transcription factors. ROS production is affected by a wide range of viruses. However, to date, the impact of viral infections has been studied only in respect to selected ROS-generating enzymes. The role of several ROS-generating and -scavenging enzymes or cellular systems in viral infections has never been addressed. In this review, we focus on the roles of biogenic polyamines and oxidative protein folding in the endoplasmic reticulum (ER) and their interplay with viruses. Polyamines act as ROS scavengers, however, their catabolism is accompanied by H_2_O_2_ production. Hydrogen peroxide is also produced during oxidative protein folding, with ER oxidoreductin 1 (Ero1) being a major source of oxidative equivalents. In addition, Ero1 controls Ca^2+^ efflux from the ER in response to e.g., ER stress. Here, we briefly summarize the current knowledge on the physiological roles of biogenic polyamines and the role of Ero1 at the ER, and present available data on their interplay with viral infections.

## 1. Introduction

Viral infections contribute significantly to human disease. Some viruses cause an acute infection that resolves spontaneously. This is the case for influenza, respiratory syncytial, rhino-, and other respiratory viruses, as well as for hepatitis A and E, measles, and dengue viruses [1]; they all trigger a wide range of symptoms including inflammation of the infected organ or cell type. Other viruses establish chronic, often life-long disease. The most prominent amongst this group of pathogens are human immunodeficiency virus (HIV), hepatitis B (HBV), C (HCV) and delta viruses (HDV), Epstein-Barr virus (EBV), human papilloma virus (HPV), and type 8 human herpes virus. Chronic viral infections are associated with various pathologies. For instance, chronic virally-induced liver hepatitis causes long-lasting liver inflammation, fibrosis, and metabolic disorders [2]. HIV causes immunodeficiency as well as neurological disorders [3]. In addition, a number of viruses are oncogenic: HBV and HCV induce hepatocellular carcinoma, EBV causes Burkett or Hodgkin lymphomas, and type 8 herpes virus causes Kaposi sarcoma [4]. As a result, both acute and chronic infections may cause permanent or persistent disability and contribute to premature mortality. Elaboration of approaches for treatment and/or prevention of virus-associated pathologies is hampered because the life cycles of these viruses and their impact on the host are not fully understood.

To date, it is clear that pathogenesis of a variety of viruses is at least partially due to altered metabolism of reactive oxygen species (ROS). ROS represent a group of relatively short-lived oxygen intermediates such as hydroxyl-radical (HO^●^), superoxide anion (O_2_^●−^), singlet oxygen (^1^O_2_), and hydrogen peroxide (H_2_O_2_). Indeed, many viruses including HBV, HCV, HIV, influenza, respiratory syncytial, rhino, dengue and tick-borne encephalitis viruses enhance ROS production in infected cells [5,6,7,8,9,10,11]. They also affect various components of the host’s antioxidant defense system. Most of the studies published on this topic so far were based on the concept of “oxidative stress” as a mere imbalance between ROS production and neutralization [12] and therefore focused on particular ROS generating and ROS-scavenging systems. In terms of ROS production, studies focused on the oxidative phosphorylation system in mitochondria, extramitochondrial NADPH-oxidases (Nox), and xanthine oxidase. However, other mitochondrial and non-mitochondrial ROS-generating enzymes and processes have been largely ignored. Amongst them are the non-respiratory mitochondrial enzymes monoaminooxidase (MAO) and α-ketoglutarate dehydrogenase (reviewed in [13]). However, here we will summarize data on two important extra-mitochondrial processes that can be important sources of ROS and affect cellular redox pathways and associated signaling. The first are biogenic polyamines, which act as ROS scavengers, even though their catabolism is associated with H_2_O_2_ production. Polyamines are involved metabolic homeostasis and developmental processes. The second source of ROS we will address is the major protein that ensures oxidative protein folding in the endoplasmic reticulum (ER): oxidoreductin 1 (Ero1). The interplay between these ROS-producing systems and viral infections will be detailed.

## 2. Biogenic Polyamines at the Crossroads of Redox Status

**Figure 1:**
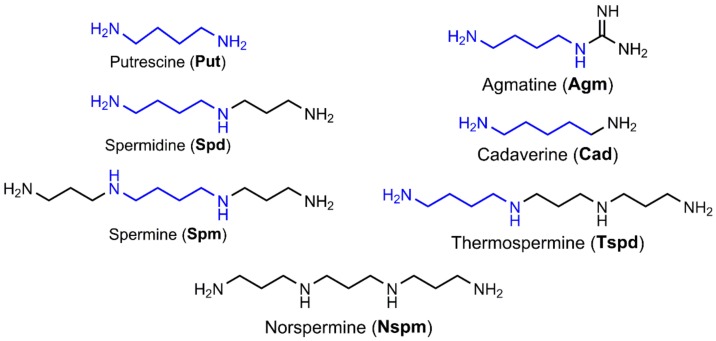
Structure of biogenic polyamines.

Biogenic polyamines are low molecular weight compounds composed of an aliphatic carbon chain with several amino groups. They include spermine, spermidine, and their precursor diamine putrescine and are present in all types of mammalian cells (Figure 1). Other species such as plants, bacteria and archaea may lack some of these polyamines but contain other members of this class of compounds [14], such as agmatine, cadaverine, thermospermine, and norspermidine (Figure 1), as well as a wide array of branched polyamines. The first polyamine identified by crystallization by Antonie van Leeuwenhoek in 1678 was spermine [15]. However, its structure was determined only in 1926 [16]. Purification of spermine from biological samples with the methods available at that time was only possible due to very high levels of polyamines in mammalian tissues and cells that can reach millimolar concentrations. In prokaryotes, polyamine concentration is estimated as 2 mM [17,18].

### 2.1. Polyamine Biosynthesis and Catabolism

**Figure 2:**
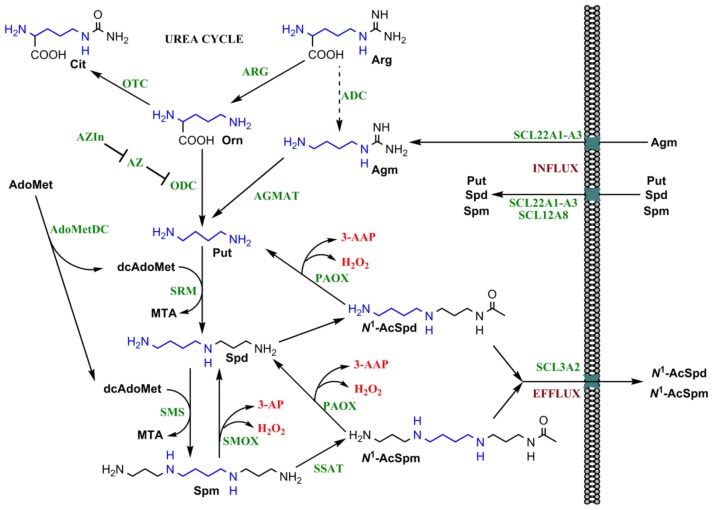
Scheme of metabolism of biogenic polyamines. Polyamines are synthesized from two metabolites of the urea cycle, namely arginine (**Arg**) and ornithine (**Orn**). Within this cycle, **Arg** is converted by arginase (ARG) into **Orn**, and the latter is transformed into citrulline (**Cit**) by an ornithine transcarbamylase (OTC). Polyamine precursor putrescine (**Put**) is synthesized from **Orn** by ornithine decarboxylase (ODC), or from **Agm** by agmatinase (AGMAT). The latter is a product of bacterial arginine decarboxylase (ADC) (a dashed arrow indicates absence of the enzyme in mammalian cells), whereas in mammals it is absorbed from the gut. Putrescine is converted into spermidine (**Spd**) and then to spermine (**Spm**) by spermidine and spermine synthases (SRM and SMS). The aminopropyl fragment is transferred by them from a decarboxylated S-adenosyl methionine (**dcAdoMet**) that is synthesized by *S*-adenosyl methionine decarboxylase (AdoMetDC). Polyamine catabolism is mediated by spermidine/spermine-*N*^1^-acetyltransferase (SSAT) with subsequent degradation of the acetylated spermine (***N*^1^-AcSpm**) and spermidine (***N*^1^-AcSpd**) by acetylpolyamine oxidase (PAOX). Spermine can also be directly converted into spermidine by spermine oxidase (SMOX). Both catabolic pathways produce toxic hydrogen peroxide and either *N*-acetyl-3-aminopropanal (3-AAP) or 3-aminopropanal (3-AP) (given in red) that are readily converted into acrolein. The bended arrows indicate utilization of a second substrate by spermine and spermidine synthases and formation of by-products during both polyamine biosynthesis and catabolism. A negative regulation of polyamine biosynthesis is achieved by antizyme (AZ) that targets ODC to proteasome, whereas functions of AZ are also inhibited by antizyme inhibitor (AZIn) (T-bar). The green abbreviations indicate enzymes and regulatory proteins. An 1,4-diaminobutyl group of polyamines deriving from arginine is marked blue.

Putrescine, a precursor of spermidine and spermine, is synthesized by decarboxylation of the amino acid l-ornithine by a pyridoxal phosphate-dependent enzyme—ornithine decarboxylase (ODC) (Figure 2) [19]. Plants and bacteria have an alternative pathway in which l-arginine is decarboxylated into agmatine by arginine decarboxylase (ADC) [20]. The latter can then be converted into putrescine by agmatinase (in *Escherichia coli*) [21], or by a combined action of agmatine deiminase and *N*-carbamoylputrescine amidase [22]. Sixteen years ago human agmatinase has been cloned suggesting that in mammalian cells putrescine can be produced from agmatine as well [23]. Indeed, agmatine is present in mammalian tissues [24,25,26,27], and has even been claimed to exhibit neurotransmitter activity [28]. Since then, several papers reported the existence of ADC in mammals, and this enzymatic activity was assigned to antizyme inhibitor 2 (AZIn2) [29,30], a regulatory protein whose place in polyamine metabolism is discussed below. However, other groups did not find evidence for a conversion of arginine into agmatine in the presence of the recombinant AZIn2 or extracts of mammalian tissues (i.e., liver) (for example, [31]). So the current view of the field is that polyamine precursor putrescine in mammals is synthesized either from arginine via ornithine, or via a reaction catalyzed by agmatinase from dietary agmatine or the one produced by gut microbes.

Spermine and spermidine are synthesized from putrescine by addition of aminopropyl groups (Figure 2). This group is taken from a decarboxylated *S*-adenosylmethionine (dcAdoMet) that is produced from *S*-adenosylmethionine by *S*-adenosylmethionine decarboxylase (AdoMetDC) [32]. The enzymes that transfer aminopropyl groups to putrescine and spermidine are known as spermidine and spermine synthases, respectively [33]. Noteworthy both ODC and AdoMetDC have cysteine residues in their active sites that readily react with nitric oxide leading to inactivation of these enzymes [34]. Nitric oxide is, therefore, a potent inhibitor of polyamine synthesis, thus forming a first link between polyamines and redox biology.

Degradation of polyamines is achieved by a combination of acetylation, oxidation, and excretion to vacuoles or extracellular fluids. In the extracellular space, polyamines are utilized by diamine oxidase and other copper-dependent extracellular enzymes [35]. In the cell spermine and spermidine undergo acetylation by spermidine/spermine-*N*^1^-acetyltransferase (SSAT) with subsequent conversion by a flavin adenine dinucleotide (FAD)—dependent acetylpolyamine oxidase (PAOX) into spermidine and putrescine, respectively [36]. Alternatively, spermine can be directly converted into spermidine by another FAD-dependent enzyme—spermine oxidase (SMOX) [37]. These extracellular and intracellular oxidases generate H_2_O_2_ and *N*-acetyl-3-aminopropanal (in case of PAOX) and 3-aminopropanal (in case of SMOX), and the latter two are readily and spontaneously metabolized into a highly toxic acrolein [38]. Noteworthy, production of these two by-products is generally associated with activity of other metabolic pathways such as lipid degradation. However, the input of polyamine-catabolizing enzymes towards ROS production is generally neglected.

SMOX and PAOX are localized in different cellular compartments: PAOX is a peroxisomal enzyme [39], whereas several different isoforms of SMOX are present in the cytoplasm and nucleus [40]. Among them, only two SMOX isoforms exhibit enzymatic activity, namely SMOX1 and SMOX5. Moreover, recently, existence of a novel SMOX-like protein in mitochondria was reported [41]. These observations may account for the greater input of SMOX compared to PAOX into production of H_2_O_2_, at least during enhanced polyamine catabolism [42]. In line with this, overexpression of SMOX leads to substantial changes in cellular redox status [43], whereas SSAT induction has no notable effect on ROS production, as revealed by commonly used ROS-sensitive dyes [42,44].

### 2.2. Regulation of Polyamine Metabolism

Intracellular levels of spermine and spermidine are maintained and tightly controlled by enzymes that catalyze rate-limiting steps of their biosynthesis (ODC) and catabolism (SMOX and SSAT) [45], as well as by their uptake [46]. Intracellular ODC and SSAT activities depend on levels of their gene transcription, translation, mRNA stability and protein degradation [47]. Expression of ODC (*ODC1*) is controlled by the transcription factors cMYC [48,49] and nuclear factor-kappa B (NF-κB) [50], whereas SSAT transcription is driven by NF-κB [51,52,53] and nuclear factor erythroid 2 (NFE2)-related factor 2 (Nrf2) transcription factors [54,55,56]. Thus, their expression is enhanced in response to inflammation, activation of phosphatidylinositol-4,5-bisphosphate 3-kinase (PI3K)/AKT [57], mitogen-activated protein kinase (MAPK) [58], and Wnt/β-catenin signaling [59], altered levels of polyamines [54,60] and enhanced ROS production [56]. Of note, the *SAT1* gene encoding SSAT contains a polyamine response element (PRE), which acts as a binding site for the classical ROS-sensitive Nrf2 factor [54,55]. Our group also demonstrated that ODC is induced in response to H_2_O_2_ via Nrf2 [56]. We did not map the binding site for this factor within the promoter, but the latter contains three TGACnnnGC sequences at −1.5, −2.1 and −4.9 kb before the transcriptional start site [61], that represent classical antioxidant response elements (ARE) to which Nrf2 is known to bind [62]. Finally, cMYC was also shown to drive transcription of genes encoding spermine synthase (SMS) and AdoMetDC [63,64].

In addition, ODC and SSAT have a very short half-life. Mechanisms of control of ODC degradation have been extensively studied and are controlled by two proteins—ODC antizyme (AZ) and antizyme inhibitor (AZIn). AZ is an inhibitor of ODC since it binds to ODC monomer and prevents assembly of the active homodimer [65]. In addition, AZ targets ODC for degradation by the 26S proteasome. These mechanisms are highly responsive to the levels of polyamines, since the active AZ is produced by a +1 frameshift of its mRNA. This frameshift is enhanced by polyamines, presumably by stabilization of a stem-loop structure in the proximity of the frameshift site. The half-life of ODC in the cell is likely also affected by ROS, since ODC can also bind to a classical Nrf2-inducible protein—NAD(P)H:quinone oxidoreductase 1 (Nqo1) [66]. Nqo1 targets ODC to the 20S proteasomal degradation pathway, which is characterized by a lower efficiency than 26S proteasomal pathway, thus prolonging the half-life of the enzyme. A second component of the system regulating ODC protein stability is AZIn. This protein, which has a structure similar to that ODC, binds to AZ more tightly than ODC. It can, therefore, displace ODC from ODC-AZ complexes or prevent their formation [65]. It should be noted that mammalian genomes contain one functional *ODC1* gene and at least four *AZ* and two *AZIn* genes that encode proteins with different expression profiles in various tissues and different properties [31,67,68].

SMOX activity is regulated only at the transcriptional level [69]. It is highly inducible by polyamine analogs and other stimuli such as ischemia-reperfusion and treatment with tumor necrosis factor alpha [37,40,51,70,71]. Increased SMOX expression was also shown to occur during differentiation of mouse myoblast C2C12 cells [72]. The other oxidase, PAOX, is generally expressed constitutively, and in most cells, this enzyme catalyzes a non-rate-limiting step [73].

Intracellular levels of polyamines are also regulated by their influx. Spermine and spermidine are imported into the cell by an active transport mechanism; however the exact transporters remain unknown. So far, several transporters have been implicated in polyamine influx and efflux. These include solute carrier (SLC) 22A1–A3 (Oct1–3), SLC12A8, SLC3A2 etc. (reviewed by Abdulhussein and Wallace in [46]). Polyamine transport is suppressed by AZ, presenting another mechanism by which antizyme reduces polyamine levels [65]. Finally, polyamines were also shown to penetrate into the cells by endocytosis [74].

### 2.3. Polyamines Can Act as Antioxidants

Although enhanced turnover of spermine and spermidine contribute to overproduction of H_2_O_2_, polyamines also contribute to the protection of the cells against ROS. Initially, it was observed that spermine and spermidine, as well as other amines, can quench ^1^O_2_^●^ [17]. Later a more detailed study from Casero’s group confirmed, that spermine indeed acts as a direct ROS scavenger [75]. Similar data were also obtained for spermidine [76], agmatine [77] as well as synthetic polyamine analogs [78]. Putrescine and cadaverine exhibit low efficacy in ROS neutralization [78,79]. Polyamines can neutralize a wide spectrum of ROS including H_2_O_2_ [76], O_2_^●−^ [78], HO^●^ [75,79,80], ^1^O_2_ [17,79], as well as synthetic radicals including 1,1-diphenyl-2-picrylhydrazyl (DPPH) radical [76] and peroxyl radicals, the latter formed from 2,2′-azo-bis-(2-amidinopropane) [78]. These studies led to the assumption that polyamines can act as bona fide ROS scavengers. However, the rate constants of the ROS scavenging reactions and therefore the input of polyamines into ROS neutralization has not been assessed in a precise manner.

Antioxidant properties of polyamines are observed at relatively high concentrations, as revealed from an example of agmatine exhibiting prooxidant activity at low (10–100 µM) and antioxidant at high (1–2 mM) concentrations [77]. At the same time, spermine exhibits protective effect towards isolated rat liver mitochondria at relatively high concentrations (25–100 mM) [81]. In particular, spermine prevents mitochondrial swelling and a decrease of mitochondrial potential triggered by the combined action of Ca^2+^ and phosphate. Moreover, polyamines bound to DNA protect the latter from ROS-induced damage [82]. This is observed at physiological (~1 mM) concentrations [82,83].

Spermidine plays a significant role in protection of cells against H_2_O_2_-induced oxidative stress [84]. Importantly, the mechanism of action of polyamines in protection of the cells against H_2_O_2_ is different from that observed for classical antioxidants like glutathione [84]. Polyamine-deficient bacteria (*Escherichia coli*) [85] or yeast cells (*Saccharomyces cerevisiae*) [86,87] exhibit more pronounced signs of oxidative stress compared to their wild-type strains. Polyamines also serve as messengers for induction of expression of antioxidant defense enzymes such as heme oxygenase 1 (HO-1) in response to e.g., β2-adrenoceptor agonists [88]. Spermine was also shown to decrease H_2_O_2_ levels and to prevent dysfunction of rat liver mitochondria [80]. The antioxidant effect of spermine at physiological concentrations in cultured myocytes is comparable to that of vitamin E [89]. In addition, at physiological concentrations spermine suppresses accumulation of free iron in cultured myocytes, with the effect being comparable to that of deferoxamine [89]. The latter may suppress generation of hydroxyl radicals via the Fenton reaction. Yeast with a defect in *spe1* gene, leading to inability to synthesize polyamines, exhibit signs of elevated ROS production, whereas supplementation of medium with spermidine reduces ROS back to the level observed in wild type cells [87]. In addition, food supplementation with spermidine decreased oxidative stress markers in aged mice [87]. Finally, such supplementation of food of *Drosophila melanogaster* confirms resistance to H_2_O_2_ and paraquat, an inducer of superoxide anion production [90]. In a zebrafish model, spermidine supplementation significantly suppressed production of ROS and NO and consequently alleviated lipopolysaccharide-triggered inflammation [91]. Altogether, these data support that polyamines contain antioxidant activity in physiological settings.

### 2.4. Polyamines in Health and Disease

Spermine and spermidine are compounds that are positively charged at physiological pH and thus bind to acidic groups of cellular macromolecules (proteins, nucleic acids or phospholipids) [60,92]. They are involved in regulation of a wide spectrum of biological processes in the cell including DNA replication and transcription, RNA processing and translation and regulation of ion channels [93,94,95]. Interaction with nucleic acids results in enhanced stability of DNA and RNA duplexes. In case of interaction with RNA, this can lead to stabilization of regulatory structural elements. Exhaustion of polyamine levels may lead to decreased expression of particular genes, as can be exemplified by decreased Heat shock protein 70 (Hsp70) [96] and cMYC [97], and cyclin-dependent kinase 4 (CDK4) [98] mRNA levels in cells treated with ODC inhibitor or in cells with decreased expression of HO-1, c-Jun and c-Fos transcription factors in response to AdoMetDC inhibition [96]. Other genes such as p21 [97] and JunD [98] are up-regulated in cells with depleted polyamine content. Since CDK4 and p21 are involved in regulation of the cell cycle, changes in their expression suggest that altered polyamine levels affect cell proliferation.

Spermine and spermidine can also affect cell fate. Monti et al. showed that HL-60 cell apoptosis triggered by 2-deoxyglucose (an inhibitor of glycolysis) can be prevented by exhaustion of the polyamine pool using ODC inhibitors, whereas addition of exogenous polyamines blocks this effect [99]. In the presence of 2-deoxyribose, polyamines do not affect cell survival rate but merely regulate the death scenario between apoptosis (high polyamine levels) and necrosis (low polyamine levels) [100]. Contrary data reported that ageing in yeast and human peripheral blood mononuclear cells (PBMCs) is accompanied by a decrease of spermidine content, elevation of oxidative stress markers and, as a result, necrotic cell death [87].

Polyamines are important for cell differentiation. For example, exogenous polyamines promote differentiation of human marrow-derived mesenchymal stem cells by regulating osteogenic and adipogenic gene expression [101]. They also affect differentiation of other types of cells including myoblasts [72], germ cells [102], and keratinocytes [103].

**Figure 3:**
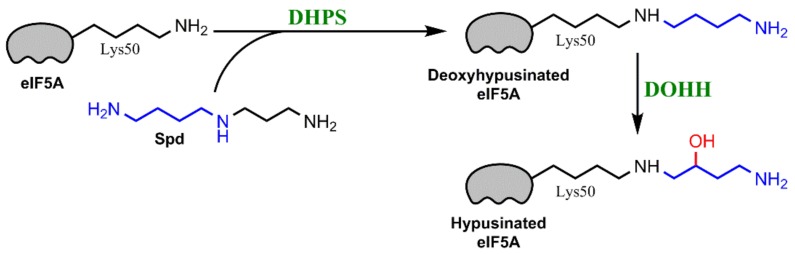
Spermidine serves as a substrate for a hypusination of eukaryotic translation initiation factor 5A (eIF5A). Lysine 50 residue of the latter is conjugated with the aminobutyl fragment from spermine by deoxyhypusine synthase (DHPS), and the resulting deoxyhypusinated factor undergoes oxidation by a deoxyhypusine hydroxylase (DOHH).

Spermidine is a precursor for a unique posttranslational modification of a lysine residue known as hypusination [104]. Hypusination has been described in the case of only one protein-eukaryotic initiation factor 5A (eIF5A) [105] (Figure 3). A mature form of eIF5A is essential for protein biosynthesis. According to the current concept, hypusinated eIF5A acts as an elongation factor [106] that allows translation bypass polyproline-rich sequences [107].

The importance of polyamines for mammalian development is supported by data from knockout mice. Animals lacking functional ODC or AdoMetDC-encoding genes are incapable of polyamine biosynthesis and die during very early embryonic stages [108]. Addition of spermidine to the culture medium during yeast/PBMCs cultivation or as a food supplement for *Drosophila melanogaster* and mice restored polyamine levels, that had decreased during ageing, counteracted ROS production and significantly prolonged lifespan [87,109,110]. Life-long food supplementation with spermidine diminished rates of occurrence of liver fibrosis and hepatocellular carcinoma in mice [110]. Investigation of the underlying mechanisms revealed that depletion of polyamines during ageing leads to activation of histone acetyl transferase (HAT) complexes and increased histone H3 acetylation [87]. Spermidine-induced longevity is promoted via histone hypoacetylation, increased expression of autophagy-related genes and, as a result, increased formation of autophagosomes [87,90,109,110]. Interestingly, these changes correlate with ROS production. Previously it was shown by several groups that oxidative stress triggered by exogenous H_2_O_2_ or ethanol in hepatocytes or lung alveolar A549 cells, induced histone acetylation through modulation of HAT complexes or their co-activators [111,112,113,114]. Similarly, autophagy is induced by ROS of various origins including mitochondria [115], ER-residing Nox4 activated by endoplasmic reticulum (ER) stress, expression of HIV transactivator (Tat)-protein [116], or treatment of cells with H_2_O_2_ [117,118]. So, spermidine might be one of the central regulators of these events.

Substantial elevation in polyamine content is also associated with pathologies including cancer and other types of cell hyperproliferation [119,120]. It is widely acknowledged that a majority of cancers exhibit increased levels of spermine and spermidine. These include liver, colon, gastric, esophagus, lung, prostate, and many others cancers (reviewed in [120]). Increased expression of polyamine-synthesizing enzymes contributes to carcinogenesis. As an example, increase in polyamine levels in skin of mice due to overexpression of ODC was accompanied by spontaneous occurrence of skin tumors both in the absence [119] and presence [121] of carcinogens. In combination with other pro-cancerous events such as expression of a mutated Ras oncogene ODC promotes invasiveness of skin cancer cells [122]. Overexpression of this enzyme in normal prostate cells is sufficient for their malignant transformation [123]. In this study, a strong up-regulation of the main polyamine enzymes (ODC, spermine oxidase, spermine synthase) and a concomitant reduction of spermine was observed in high-grade prostate intraepithelial neoplasia (HGPIN) tissues as compared to normal prostate glands with the expression levels for SMOX expression being the highest. Elevated polyamine levels also promote angiogenesis, which is one of the tumors hallmarks [124]. So, enhanced polyamine biosynthesis can be an early event in tumorigenesis, and its combination with other oncogenic events may result in formation of tumor cells.

Alteration of polyamine metabolism also contributes to bacteria-induced tumorigenesis in the gastrointestinal tract. Wilson’s group clearly demonstrated that development of gastric cancer during *Helicobacter pylori* infection is due to induction of spermine oxidase [125,126]. The procarcinogenic effect of this enzyme is at least partly associated with production of H_2_O_2_, since its expression and concomitant development of tumors were strongly associated with levels of H_2_O_2_ and oxidative DNA damage. Similar findings were also reported for the enteric bacterial strain *Bacteroides fragilis* [127,128].

### 2.5. Polyamines and Viral Infections

Data on the interplay between polyamines and viral infections are controversial. The majority of them were published in the 1970s–1980s, when several key players of their metabolism were just discovered, but the technical approaches for their investigation not yet elaborated. These old data refer mostly to incorporation of polyamines in virions, and to sensitivity of virus replication to inhibition of polyamine biosynthesis.

Since spermine and spermidine can bind to DNA and RNA, it is not surprising that they were found in viral particles of various viruses. Virions from densonucleosis viruses 1 and 2 contain putrescine, spermidine, and spermine comprising 1.41% (*w*/*w*) of the virus particle and neutralizing 26% of the single-stranded viral DNA [129]. Vaccinia virus of the *Poxviridae* family [130] and bacteriophage R17 [131] also incorporate polyamines into virions. Encephalomyocarditis virus contains approximately 200 molecules of putrescine, 100 molecules of spermidine, and 40 molecules of spermine that neutralize 11% of the negative charge of the viral genome [132]. Herpes simplex virus (HSV) also contains both spermine and spermidine, which are compartmentalized in capsid and envelope, respectively [133]. Presence of polyamines was also shown for influenza virus (in which they neutralize approximately 38% of RNA phosphates) [134] and rhinovirus [135]. Other viruses such as Semliki Forest virus do not contain polyamines in their capsids [136].

Both old and recent papers suggest that polyamines are crucial for replication of many viruses. Inhibition of polyamine biosynthesis inhibits replication of a variety of RNA viruses belonging to the families *Picornaviridae*, *Flaviviridae*, *Coronaviridae*, *Filoviridae*, and *Buniaviridae* [137,138,139] as well as of DNA-containing HSV [140], human cytomegalovirus (HCMV) [141,142,143], and vaccinia viruses (Tyms et al. 1983). However, the mechanisms by which polyamine depletion suppresses their replication is likely to be different. In the case of HCMV, polyamines are required for virion assembly, either at the level of DNA packaging or capsid envelopment [141]. For HSV, arboviruses, and poxviruses, polyamines are needed for viral genome replication [130,138,140]. Another mechanism by which polyamines contribute to viral replication is maintaining hypusination of eIF5A factor, as shown for Ebola and Marburg viruses [139] and human immunodeficiency virus (HIV) [144]. In HIV-infected cells, hypusination inhibitors suppress viral gene expression at the level of transcription initiation [144]. Finally, induction of SSAT and a concomitant decrease in polyamines were shown to inhibit replication of Zika and chikungunya viruses [138]. The same effect was observed for both pathogens when biosynthesis of polyamines was blocked [138]. Depletion of polyamines affected both, viral genome replication and protein biosynthesis. Interestingly, passaging chikungunya virus in the presence of increasing d,l-difluoromethylornithine (DFMO) concentrations led to establishing clones resistant to polyamine depletions [145]. The latter was associated with mutations in the non-structural protein 4 (*nsP4*) gene that enhanced methylation of viral genomes and their binding to cell membranes.

The least studied is the effect of viral infection on polyamine metabolism itself. Cells infected with HSV-1 exhibit decreased levels of spermine and spermidine, presumably due to suppressed polyamine biosynthesis [146,147]. In addition, HSV-2 was shown to suppress subsequent steps of polyamine biosynthesis, i.e., conversion of putrescine into spermidine and then to spermidine [148] At the same time, HCMV, that also belongs to the *Herpesviridae* family, triggers pronounced induction of ODC [149] and a concomitant increase in spermine and spermidine levels [142,150]. However, other authors noted, that elevation of putrescine and spermidine levels was accompanied by reduced levels of spermine [151]. The latter implies induction of SSAT and/or SMOX, however, expression of the former was not studied, whereas the latter was not yet discovered at the time. Transient increase in ODC activity was also shown for cells infected with vaccinia virus [152]. Some data also suggest inhibited polyamine biosynthesis by human adenovirus type 5 [152]. In the lymphocytes from HIV-1-infected patients an increase in polyamine levels and SSAT activity was reported [153], though such changes do not occur in the infected lymphocytes in vitro [154]. Several years ago, HIV Tat protein was shown to induce SMOX, which plays role in virally-induced neurotoxicity [155]. Finally, our group recently reported that transient expression of HCV core and nonstructural protein (NS) 5A (NS5A) proteins leads to activation of polyamine metabolism and elevation of intracellular polyamine levels [156]. However, long-lasting expression of viral proteins in the context of the full length replicon resulted in a decrease in spermine and spermidine, accompanied by an induction of spermine oxidase. Spermine oxidase may be required for efficient replication of the viral genome, since polyamines were previously shown to inhibit helicase activity of HCV NS3 protein [157]. Finally, *Chlorella* virus that infects some algae species encodes enzymes of polyamine biosynthesis itself [158,159]. However, similar examples are not known for mammalian viruses.

## 3. ER Oxidoreductins as Key Players in Oxidative Protein Folding, Calcium Efflux from the ER, and Evasion of Cancer Cells from Immune Responses

### 3.1. Ero1 Is the Key Player in Oxidative Protein Folding

**Figure 4:**
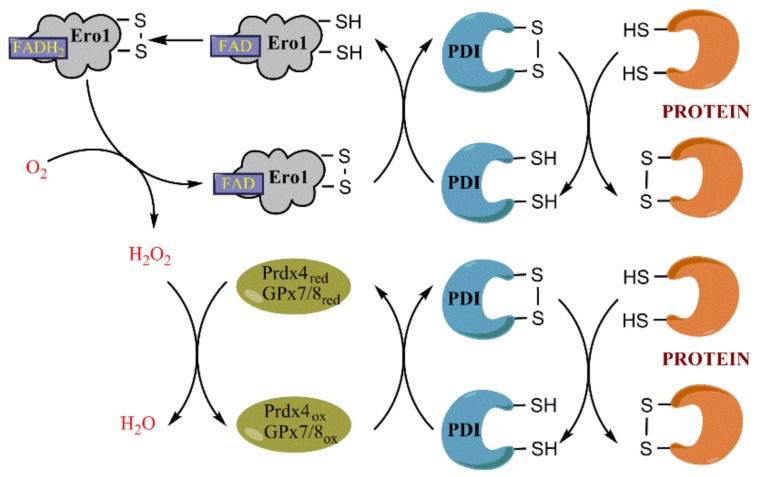
Role of ER oxidoreductin 1 (Ero1), peroxiredoxin 4 (Prdx4) and glutathione peroxidases (GPx) 7 and 8 in oxidative protein folding. The primary oxidative equivalents are generated by a FAD-dependent enzyme Ero1 that reduces molecular oxygen (O_2_) into hydrogen peroxide (H_2_O_2_). It leads to formation of a disulfide bond between two cysteines of Ero1 followed by a disulfide-dithiol exchange with a protein disulfide isomerases (PDI) and later with the target protein. Noteworthy, the figure does not show exchanges between several pairs of cysteines within Ero1. The generated H_2_O_2_ is utilized by Prdx4 or GPx7/8, that also oxidize PDI. Thus, a reduction of one oxygen molecule generates equivalents for oxidation of two pairs of cysteines of a target protein.

The endoplasmic reticulum is an organelle that ensures production and folding of secreted, plasmatic, Golgi, and lysosomal proteins, that serves as intracellular storage of calcium ions and as site for lipid synthesis. Protein folding involves formation of disulfide bonds between cysteine residues of the same protein or between two different proteins. Disulfide bonds are introduced into the nascent polypeptide via dithiol-disulfide exchange reaction with assistance of oxidoreductases including protein disulfide isomerase (PDI), ERp44, ERp46, ERp72 and other members of the PDI family [160] (Figure 4). PDI is also capable of reducing incorrect S–S bonds formed spontaneously, thus allowing subsequent formation of the correct disulfide bonds [161]. Specifically, PDI family members form mixed disulfides with the client proteins, with subsequent release of oxidized substrates and reduced PDIs [162,163]. Regeneration of PDI into its oxidized form is tightly linked to H_2_O_2_ production or scavenging.

The primary oxidation equivalents for protein folding are generated by an enzyme referred to as ER oxidoreductase (Ero1). It was originally identified in yeast in 1998 as the ER-residing glycosylated enzyme Ero1p, and shown to be involved in oxidative protein folding [164,165]. In 2000, two human orthologs were found and termed Ero1-like (Ero1L) α [166] and β [167]. Ero1α is highly expressed in esophagus, liver, heart, stomach, duodenum, and placenta, whereas Ero1β is expressed mostly in the pancreas and at lower levels in the stomach and testis [167,168]. Ero1β exhibits a two-fold higher enzymatic activity compared to Ero1α [169].

Both yeast and mammalian ER oxidoreductins transfer electrons from PDI to molecular oxygen, leading to its reduction into H_2_O_2_ [170,171]. Ero1 is a flavoprotein, so the reaction is dependent on levels of FAD [170,171,172]. Reduction of O_2_ is accompanied by formation of an intramolecular disulfide bond between Cys352 and Cys355 of the enzyme (the numbers correspond to positions of amino acids within yeast Ero1p) [173]. This disulfide bond is later passed to another pair of cysteines (Cys100 and Cys105) within Ero1 and the resulting disulfide bond is cleaved during formation of an intermolecular disulfide with PDI and subsequent regeneration of the latter [174]. Other intramolecular disulfide bonds also exist that are implicated in regulation of Ero1p activity [175]. Similar intra- and intermolecular disulfide-dithiol exchange has been demonstrated for human Ero1α and Ero1β isoforms [176,177,178]. Kinetic analysis revealed that formation of the correct regulatory S–S bond within Ero1α in the presence of O_2_ or H_2_O_2_ is slow, but is significantly enhanced in the presence of PDI family members [179]. Again, enzymatic activity of the mammalian oxidoreductase is regulated by several additional pairs of cysteines. For example, a disulfide bond between Cys208 and Cys241 residues of Ero1α forms a seal for FAD in the site of molecular oxygen reduction, and its reduction by PDI leads to release of FAD and, therefore, to inactivation of the enzyme [176]. Substitution of some of these cysteine residues results in a constantly active Ero1 enzyme, i.e., in a constituent H_2_O_2_ production [180]. It is also worth noting, that PDI is the major but not the sole substrate of Ero1α and Ero1β [169,174,181], they can also oxidize other members of PDI family such as ERp46, ERp57, and ERp72 [179,181,182,183]. Several PDI family members are also implicated in retention of Ero1α/β in the ER [184,185].

Before identification of Ero1, protein oxidation folding was thought to be mediated by oxidized glutathione (GSSG). However, later it was found that GSSG is not required [164]. In contrast, reduced glutathione (GSH) can interact with oxidized PDI, leading to reduction of the latter and formation of GSSG [186,187]. Since the reduced PDI activates Ero1, increase in GSH content in the ER enhances H_2_O_2_ production [188]. Recently, a negative feedback loop was described: GSH-induced Ero1α activation results in massive production of H_2_O_2_, leading to suppressed GSH import into ER [189]. On the other hand, absence of GSH would result in hyperoxidation of proteins [186]. So, a balance between GSH and GSSG in the ER lumen forms a sort of a “buffer” for oxidative protein folding, that allows efficient isomerization of S–S bonds and conversion of non-native disulfides into native ones [182,187,190,191].

### 3.2. Regulation of Ero1 Expression

Expression of yeast Ero1p and its mammalian orthologs Ero1α and Ero1β is not constitutive. ER stress and concomitant unfolded protein response (UPR) cause significant increase in Ero1p/Ero1β and moderate increase in Ero1α levels [164,165,167,192,193,194]. Specifically, Ero1α is induced by the protein kinase R (PKR)-like ER kinase (PERK)/CCAAT/enhancer-binding protein homologous protein (CHOP) pathway of the UPR [193,194]. Another factor that plays an even greater impact on Ero1α expression is the level of O_2_, as hypoxia leads to a profound overexpression of this oxidoreductase via induction of hypoxia-inducible factor 1α (HIF-1α) [192,195,196]. In addition, Ero1α is upregulated in response to depletion of iron content, triggered, for example, by the chelating agent deferoxamine [192]. Finally, induction of Ero1α and other UPR-responsive genes has been described during nitrosative stress, and the underlying mechanism involved *S*-glutathionylation of PDI cysteine(s) [197].

### 3.3. Role of Prdx4 and GPx7/8 in Scavenging H_2_O_2_ in the ER and in Protein Folding

Since oxidative protein folding is accompanied by constant production of H_2_O_2_ in the ER, intensive search identified several lumenal enzymes, that efficiently scavenge H_2_O_2_. These include the ER-residing peroxiredoxin 4 (Prdx4) [198] and glutathione peroxidases 7 (GPx7) [199] and 8 (GPx8) [200]. However, the exact role of each of these factors is still not fully understood. Initially it was proposed that Prdx4 is the bona fide scavenger of H_2_O_2_ generated by Ero1α, as the levels of peroxide production correlated to hyperoxidation of this peroxiredoxin, which also plays a cytoprotective role [198]. However, Appenzeller-Herzog’s group suggested that Prdx4 is implicated in neutralization of H_2_O_2_ only in the settings of constantly active Ero1α, whereas in cells expressing the wild type enzyme the major antioxidant role is attributed to GPx8 [200]. Noteworthy, the latter efficiently prevents leakage of H_2_O_2_ from the ER to the cytoplasm [200]. At the same time Ero1α is controlling luminal levels of hydrogen peroxide, that correlate with the expression levels of this enzyme [201].

Recent data suggest, that this scavenging of H_2_O_2_ is not a mere antioxidant reaction, but an element of oxidative protein folding. All these three enzymes contribute to the formation of disulfide bonds. It was proposed that both GPx7 and GPx8 are not the real glutathione peroxidases but protein disulfide isomerase peroxidases, that enhance peroxidase activity of PDI family members [202]. Indeed, they interact with Ero1α, thereby enhancing its enzymatic activity and concomitant oxygen consumption [202], and with PDI [199], thus forming an Ero1α/PDI/GPx7/8 triad that drives protein folding. Prdx4 also interacts with and oxidizes PDI [203,204,205] and other members of its family such as ERp56 and P5 [161]. Interestingly, Prdx4 drives peroxide-dependent oxidative protein folding even in cells lacking Ero1α, thus suggesting existence of alternative, yet undiscovered, sources of H_2_O_2_ in the ER lumen [204]. To sum up, these antioxidant enzymes allow formation of two disulfide bonds at a cost of consumption of one oxygen molecule, without affecting the redox status in the ER lumen.

### 3.4. Ero1 Controls Efflux of Calcium Ions from the ER

Redox biology is tightly linked to homeostasis of calcium ions in the cell. In most compartments the concentration of Ca^2+^ is low (~10^−7^ M), whereas the ER acts as a storage for calcium ions [206]. An estimated concentration of Ca^2+^ in the ER lumen is ~10^−4^ M [206]. Thus, it is not surprising that alteration of normal ER functioning may result in efflux of Ca^2+^ from the ER, yielding a significant increase in calcium levels in the cytoplasm and mitochondria and causing a wide spectrum of events. More and more data describe Ero1 as one of the key regulators of calcium homeostasis.

Elevated Ca^2+^ levels in the ER, compared to other organelles, are maintained by sarco/endoplasmic reticulum Ca^2+^-ATPase (SERCA). SERCA mediates uptake of Ca^2+^ and controls activity of inositol 1,4,5-trisphosphate receptor (IP3R) and ryanodine receptor (RyR) that mediate Ca^2+^ efflux [206]. There are three isoforms of SERCA: SERCA1 is expressed in the skeletal muscle, SERCA3—Primarily in cells of hematopoetic origin and some epithelial cells, whereas SERCA2 is expressed ubiquitously [207]. Since one of the SERCA2 cysteines undergo *S*-glutathionylation [208], it is tempting to speculate, that the status and level of Ero1 may affect the functioning of this pump and, therefore, calcium homeostasis. Indeed, the mutant of the target cysteine residue leads to Ca^2+^ depletion of the ER stores as well as in altered Ero1α induction in response to hypoxia [209]. Since decrease in Ca^2+^ levels in the ER is known to down-regulate SERCA2 expression [210], this would amplify the signal. SERCA2 is also known to be sensitive to inactivation due to hyperoxidation of its cysteine residue(s) [211]. However, the pump is protected from hyperoxidation by selenoprotein N (SEPN1), which interacts with SERCA2. Moreover, increased expression of Ero1α is accompanied by induction of SEPN1, which protects the pump from the hazardous effects of the oxidoreductase [211]. However, it could be assumed, that any other factor that targets this selenoprotein may lead to altered calcium homeostasis.

Ero1α also affects calcium storage by interaction with the IP3R. The latter is localized at mitochondria-associated membranes (MAMs), i.e., at the site of physical interaction between ER and mitochondria [212]. IP3R interacts with voltage-dependent anion channel (VDAC) through Grp75, whereas VDAC acts as a channel for Ca^2+^ and ROS in the outer membrane of mitochondria [213]. Activation of IP3R leads to efflux of calcium ions from the ER and their accumulation in mitochondria [166]. Ca^2+^ influx through the inner mitochondrial membrane is mediated by mitochondrial calcium uniporter channel (MCU). Interestingly, MCU regulatory protein termed mitochondrial calcium uptake 1 (MICU1) is activated when calcium concentrations reach ~2–10 μM [214]. While efflux of Ca^2+^ from the ER results in <0.8 μM general concentration in the cytoplasm, the local concentration at MAMs reaches the target values, thus allowing a direct flux from the ER to mitochondria [214,215]. Ero1α is also localized at MAMs under normal oxidizing ER conditions, whereas reducing agents and hypoxia lead to its relocalization [216]. In 2012, Sitia’s group revealed that Ero1α controls efflux of Ca^2+^ from the ER to mitochondria, and its induction leads to decreased levels of the ions in the ER, their accumulation in the mitochondrial matrix and concomitant mitochondrial dysfunction [217].

Both SERCA2 and IP3R are also regulated by PDI family members. ERp44 was shown to inhibit Ca^2+^ efflux by IP3R through direct interaction with either of them. This interaction was disrupted during saturation of ER Ca^2+^stores [218]. ERp57, in contrast, suppressed activity of SERCA2b, which resulted in increased periods and length of Ca^2+^ oscillations [219]. Currently, no direct data exist on the effect of Ero1α on these interactions. However, it can be assumed that overexpression of this oxidoreductase, which leads to changes in redox status of members of the PDI family, may constitute an additional mechanism of Ca^2+^ efflux from the ER. This is consistent with evidence that shows that ER stress leads to calcium release from the ER through Ero1α-mediated IP3R activation [194].

### 3.5. Role of Ero1 in Pathology

Ero1α is associated with carcinogenesis and other pathologies. It has been clearly established that breast [220], gastric [221], esophagus [222], and colon [222,223] tumors exhibit significantly higher levels of Ero1α expression compared to the respective normal tissues. Moreover, levels of its expression correlate with rates of recurrence of the disease and with reduced survival rates [220,221,224]. Silencing Ero1α inhibits growth and metastatic potential of breast cancer cells [220]. To our knowledge, there are no data about interference of this enzyme with the cell cycle machinery, proapoptotic factors or tumor suppressors. At the same time, several lines of evidence suggest, that Ero1α facilitates evasion of tumor cells from host immune responses and tumor vascularization. Tanaka et al. showed that Ero1α plays important roles in production of vascular endothelial growth factor (VEGF), with a significant correlation between expression of the oxidoreductase and angiogenesis in breast tumors [225]. The same group also reported that overexpression of Ero1α in human breast cancer up-regulates programmed cell-death 1 ligand 1 (PD-L1) [226], a well-known regulator of an immune checkpoint [227]. Mechanistically, this was due to enhanced oxidative protein folding and by induction of PD-L1 transcription. Production of PD-L1 by tumor cells promotes their escape from immune responses by triggering death of T-cells [228]. Another mechanism by which Ero1α contributes to evasion of tumor cells from immune responses is by promoting infiltration of myeloid-derived suppressor cells (MDSCs) into the tumor [229]. The latter results from enhanced production of granulocyte colony-stimulating factor (G-CSF), chemokine (C-X-C motif) ligand (CXCL) 1 and 2 cytokines/chemokines. MDSCs exhibit immunosuppressive activity by preventing activation of T-cells [230]. Therefore it is not surprising, that mice with Ero1α knock-out are characterized by slower tumor growth [229]. However, other groups revealed that ER oxidoreductin 1α can also enhance T-cell responses. It was exemplified by demonstrating that Ero1α is involved in oxidative folding of major histocompatibility complex 1 (MHC-1) [223], that is crucial for interaction with cytotoxic CD8^+^ T-cells [231]. Indeed, colon carcinoma cells exhibit elevated expression of Ero1α compared to normal cells, and in these cells, oxidative MHC-1 folding is enhanced [223]. Moreover, expression of Ero1α and concomitant MHC-1 folding are enhanced in hypoxic conditions, as shown for both colon cancer and thymoma cells [223,232]. As a result, Ero1α-overexpressing cells trigger an enhanced cytotoxic T-cell response, whereas Ero1α down-regulation inhibits the response, and hypoxia partially restores it. The discrepancy between pro- and antitumor activity of Ero1α is unknown.

### 3.6. Ero1α in Viral Infections

Data on the status of Ero1α in human viral infections are scarce and limited to just two human and one plant virus. Most of the data concern HCV. HCV is known to trigger massive ROS production in the infected hepatocytes, both in vitro and in the context of the liver (reviewed in [233]). Amongst the ten viral proteins, two are considered as main inducers of ROS: core and NS5A [234,235,236]. For a long time, it has been considered, that ROS production in HCV core-expressing cells was enhanced primarily in mitochondria and resulted from induced activity of mitochondrial Ca^2+^ uniporter [237] or interaction of the viral protein with mitochondrial chaperone Hsp60 [238]. In addition, HCV core was shown to cause UPR stress, leading to depletion of ER calcium stores [239]. We demonstrated, that HCV core up-regulated expression of Ero1α, and that prevention of its induction not only reduced H_2_O_2_ levels in the cytoplasm, but also blocked mitochondrial Ca^2+^ uniporter-mediated ROS production in mitochondria [240]. In contrast, Ero1α expression was not altered in cells overexpressing NS5A [241], another HCV protein that was considered to up-regulate ROS production via alteration of calcium homeostasis [234]. Nevertheless, Ero1α is indeed one of the key players in HCV-induced redox alterations, whereas its role in the viral life cycle still remains to be uncovered.

Chikungunya virus is another pathogen that is thought to cause oxidative stress in infected cells [242]. Nothing is known about the underlying molecular mechanisms and the potential involvement of Ero1α. However, recent data suggest, that Ero1α is important for the chikungunya viral life cycle, since its inhibitor EN460, as well as inhibitors of PDI, suppress viral replication, especially during its early stages [243].

Finally, a recently published data suggest, that Ero1 is a crucial component of replication of a plant brome mosaic virus (BMV) [244]. This oxidoreductase facilitates formation of a disulfide-linked complex of viral replication protein 1a protein, thus driving capping of BMV RNA. In addition, Ero1p triggers leakage of oxidizing equivalents from ER lumen. The latter is likely to contribute to enhancement of pathogen replication, as both plant and human viruses are known to exploit ROS for efficient capping and synthesis of their genomes [245,246].

## 4. Interplay between Polyamine Metabolism and ER Stress/Unfolded Protein Response

In the current literature, polyamine metabolism and oxidative protein folding in the ER are still being considered as separate events, even though tight interlinks have been reported between them. It has been known for a long time that polyamine depletion, resulting from their inhibited biosynthesis, causes swelling of ER [247]. Prunotto et al. provided evidence for increased expression of the classical UPR markers Grp78 and Grp94 in MDCK cells treated with profibrotic transforming growth factor β1 (TGFβ1) and DMFO, an inhibitor of polyamine biosynthesis, compared to TGFβ1-treated cells [248]. This effect was due to depletion of polyamines, as exogenous putrescine, spermidine, and spermine prevented expression of Grp78 and Grp94. Unfortunately, this study presented no data on expression of UPR marker genes in TGFβ1-untreated cells and did not study oxidative protein folding in the ER. This gap was later filled in by Kahana’s group, who studied changes in gene expression in NIH 3T3 cells treated with DFMO using Affimetrix microarrays [249]. They revealed, that polyamine depletion leads to specific activation of the PERK pathway, but no changes in expression of genes regulated by the other two branches of the UPR. At the same time, the microarray dataset demonstrated pronounced induction of protein disulfide isomerase A6 (PDIA6) and peroxiredoxin 4. Since the latter factor is a key component of the oxidative protein folding machinery, and Ero1α expression is regulated by the PERK-activating transcription factor 4 (ATF4)-CHOP pathway, that is activated in these cells (see above), this finding suggests existence of an interplay between polyamines and protein folding. Moreover, a depletion of ER calcium stores observed in DFMO-treated cells further supports this observation, taking into account the known role of Ero1α in Ca^2+^ homeostasis.

Similar changes occur in response to activation of polyamine catabolism. In rats with acute pancreatitis, due to conditional overexpression of SSAT, pancreatic acinus cells exhibited pronounced signs of endoplasmic reticulum dilatation soon after activation of polyamine catabolism [250]. At the same time, it was shown that SSAT overexpression and concomitant polyamine depletion trigger mitochondrial dysfunction leading to cell death [251]. Several papers report, that enhanced polyamine catabolism interferes with exogenous and endogenous gene translation [252,253] and triggers ER stress and UPR. The latter was demonstrated in various systems based on cell lines overexpressing SSAT [254] and animals with induced SSAT in response to cisplatin [254]. Translation inhibition was caused by depletion of polyamines, since restoration of polyamine content in SSAT-overexpressing cells with spermine and spermidine as well as their mimetics restored translation [253]. The observed ER stress was probably triggered by toxic by-products of spermine and spermidine degradation, as both ER stress as well as concomitant cell death could be prevented by compounds, that scavenge aldehydes alone or in combination with pegylated catalase [254]. The status of oxidative protein folding was not accessed in any of these studies. One could speculate that induction of SSAT does not lead to induction of Ero1α. Indeed, SSAT1 targets HIF-1α, which controls Ero1α expression for degradation [195]. However, Ero1α could still be induced by the UPR PERK pathway [194], which is induced by SSAT overexpression.

ER stress can also result from dysregulated eIF5α hypusination. In a yeast model, Rossi et al. found that eIF5A is involved in cotranslational translocation of the forming polypeptide into the ER, and that defects in hypusination of this factor lead to induction of several stress-induced chaperones [255]. Park’s group revealed that depletion of eIF5α from HeLa cells does not affect overall translation, but specifically upregulates many genes of the UPR [256]. Interestingly, eIF5α depletion did not affect translation of genes encoding proteins containing polyproline sequences. Indeed, the majority of dysregulated proteins did not possess any polyproline stretches. Again, this study did not investigate expression of Ero1α. However, the provided data set clearly showed increase in expression of several key ER chaperones and proline-disulfide isomerases as well as of mitochondrial chaperones [256]. Changes were also found in expression of VDAC1 and VDAC3, which are involved in maintaining calcium homeostasis. Therefore, it is tempting to speculate, that affecting this part of polyamine metabolism may affect cellular redox status by altering oxidative protein folding in the ER. However, this assumption needs to be validated experimentally.

Polyamines not only modulate proper ER functioning, but also affect processes downstream of calcium efflux from the ER. Indeed, overexpression of ODC has been reported to protect cells from death triggered by thapsigargin (Tg) [257], a widely known non-competitive inhibitor or SERCA. This agent depletes ER Ca^2+^ stores, thus inducing ER stress, subsequent accumulation of calcium ions in mitochondria and, as a result, apoptosis. ODC overexpression in Tg-treated cells prevented induction of UPR-responsive genes and decrease in SERCA levels, suppressed increase in intramitochondrial Ca^2+^ concentration, mitochondrial membrane depolarization, release of cytochrome c and other preapoptotic events [257]. Induction of UPR-inducible proapoptotic genes (i.e., CHOP) at the translational level and subsequent apoptosis could also be prevented by inhibition of deoxyhypusine synthase with its inhibitor GC7, as was shown in a pancreatic β-cell line treated with Tg [258]. This provides additional evidence that polyamine metabolism may affect ER homeostasis, and changes in spermine and spermidine levels can affect protein folding in the ER and cause mitochondrial dysregulation. Thus, mitochondrial dysfunction and production of ROS in this organelle, observed during many viral infections, may not necessarily be directly induced by a virus but can also occur as a secondary or even tertiary event.

Most of the data discussed in this chapter concern uninfected cells or animal studies, while few data based on infectious models are discussed. This does not mean that the same interlinks do not exist in the context of viral infections. Indeed, our group revealed that hepatitis C virus alters polyamine metabolism specifically by inducing the two catabolic enzymes SSAT and SMOX in a transient and persistent fashion, respectively [156]. This effect was mediated by two viral proteins: core and NS5A [156]. These two proteins are known to trigger ROS production in infected cells [234,235,259]. In addition, HCV core, the viral protein that displays the highest prooxidant activity [235], upregulates expression of Ero1α, and this event was found to be upstream to core-triggered superoxide production in mitochondria, as well as to accumulation of hydrogen peroxide in cytoplasm [240]. This clearly shows that polyamine-metabolizing enzymes and oxidative protein folding in ER can (i) significantly contribute to virus-induced oxidative stress, (ii) generate ROS outside mitochondria, and (iii) regulate proper functioning of this organelle. Other examples come from studies of parasitic infections and from plant viruses. It is acknowledged that Arabidopsis thaliana, like many other species, defend against pathogens by inducing an event referred to as hypersensitive response (HR). Its induction during infection with mosaic cucumber virus is accompanied by enhanced polyamine biosynthesis [260]. Detailed analysis of changes in gene expression between control plants and those treated with spermine revealed induction of UPR [261] and in particular of AtbZIP60 [260]—one of its key regulators [262]. Given that AtbZIP60 controls expression of several protein disulfide isomerases (i.e., AtPDI6, AtPDI9, AtPDI10, and AtPDI11) [263], these facts provide another line of support of a tight interplay between polyamine metabolism and oxidative protein folding in the ER. Finally, Yarlett’s group performed analysis of changes in HTC-8 cells infected with the Cryptosporidium parvum parasite [264]. They revealed (i) perturbation of polyamine metabolism (i.e., induction of SSAT and ODC leading to changes in the ratio between individual polyamines and accumulation of acetylated spermine and spermidine), (ii) signs of UPR and (iii) enhanced H_2_O_2_ production and concomitant activation of the antioxidant Nrf2/ARE pathway.

## 5. Conclusions

Despite a tremendous significance of polyamines and Ero1α for normal cell growth, their interplay with viral infections remains poorly studied. However, there are several key facts that merit further investigations. First, various groups demonstrated that inhibitors of polyamine biosynthesis exhibit antiviral activity against a wide spectrum of DNA and RNA viruses. However, there is only limited data unveiling changes in polyamine levels and levels of expression of polyamine-metabolizing enzymes in infected cells. Should these scarce data hold true, these changes could contribute to various virus-associated pathologies such as tissue damage and neoplastic transformation in chronic infections, or barrier dysfunction and concomitant susceptibility to bacterial pathogens in respiratory viral infections. Secondly, it has been clearly shown that polyamine catabolism is associated with production of H_2_O_2_ as a by-product. At the same time, with the exception of HIV, it is not known if augmented ROS production in infected cells is due to SMOX or PAOX. Again, SMOX-derived peroxide and acrolein could be partially responsible for the pathogenicity of these infections. Third, the alternative polyamine biosynthesis pathway requires further studies, both in normal and infected cells. Fourth, the effect(s) of viruses on the ER oxidative protein folding machinery also requires future studies. Since Ero1α is induced in response to UPR and hypoxia, it may turn out that many infections that trigger ER stress and induce HIF-1α up-regulate its expression. If this is the case, this might be the upstream cause for many well-characterized events in infected cells, such as disrupted calcium homeostasis, mitochondrial dysfunction and changes in redox status of the cells in total and in the ER in particular. Moreover, for HCV, which induces GPx8 proteolysis, induction of Ero1α could lead to leakage of H_2_O_2_ from the ER. Thus, the interplay between viruses and the ER oxidative folding machinery can contribute to infection-associated pathologies. In conclusion, a significant amount of data demonstrate ER stress, altered protein folding and dysregulated polyamine metabolism in infected cells, but the molecular links between these events and their respective roles in the cellular stress and pathophysiology associated with infection will require further studies.

## Abbreviations

ROS	Reactive oxygen species
ER	Endoplasmic reticulum
Ero1	ER oxidoreductin 1
Ero1L	Ero1-like
HIV	Human immunodeficiency virus
HBV	Hepatitis B virus
HCV	Hepatitis C virus
HDV	Hepatitis D virus
EBV	Epstein-Barr virus
HPV	Human papilloma virus
Nox	NADPH-oxidase
MAO	Monoaminooxidase
ODC	Ornithine decarboxylase
ADC	Arginine decarboxylase
AZInI	Antizyme inhibitor
ARG	Arginase
OTC	Ornithine transcarbamylase
AGMAT	Agmatinase
SRM	Spermidine synthase
SMS	Spermine synthase
3-AAP	*N*-acetyl-3-aminopropanal
3-AP	3-Aminopropanal
dcAdoMet	Decarboxylated *S*-adenosylmethionine
AdoMetDC	*S*-Adenosylmethionine decarboxylase
SSAT	Spermidine/spermine-*N*^1^-acetyltransferase
FAD	Flavin adenine dinucleotide
PAOX	Acetylpolyamine oxidase
SMOX	Spermine oxidase
DHPS	Deoxyhypusine synthase
DOHH	Deoxyhypusine hydroxylase
NF-κB	Nuclear factor-kappa B
Nrf2	nuclear factor erythroid 2 (NFE2)-related factor 2
PI3K	Phosphatidylinositol-4,5-bisphosphate 3-kinase
MAPK	Mitogen-activated protein kinase
SLC	Solute carrier
PRE	Polyamine response element
ARE	Antioxidant response element
AZ	Antizyme
Nqo1	NADPH:quinone oxidoreductase 1
PERK	Protein kinase R (PKR)-like ER kinase
CHOP	CCAAT/Enhancer-Binding Protein Homologous Protein
ATF4	Activating transcription factor 4
HIF-1α	Hypoxia inducible factor 1α
G-CSF	Granulocyte colony-stimulating factor
CXCL	Chemokine (C-X-C motif) ligand
FDMO	d,l-difluoromethylornithine
DPPH	1,1-Diphenyl-2-picrylhydrazyl
PBMC	Peripheral blood mononuclear cells
eIF5A	Eukaryotic translation initiation factor 5A
HAT	Histone acetyl transferase
HSV	Human herpes virus
HCMV	Human cytomegalovirus
PDI	Protein disulfide isomerase
GSSG	Oxidized glutathione
GSH	Reduced glutathione
UPR	Unfolded protein response
Prdx4	Peroxiredoxin 4
GPx	Glutathione peroxidase
SERCA	Sarco/endoplasmic reticulum Ca^2+^-ATPase
IP3R	Inositol 1,4,5-trisphosphate receptor
RyR	Ryanodine receptor
SEPN1	Selenoprotein N
VDAC	Voltage-dependent anion channel
MCU	Mitochondrial calcium uniporter channel
MICU1	Mitochondrial calcium uptake 1
MAMs	Mitochondria-associated membranes
PD-L1	Programmed cell-death 1 ligand 1
MDSCs	Myeloid-derived suppressor cells
MHC-1	Major histocompatibility complex 1
